# R269C variant of ESR1: high prevalence and differential function in a subset of pancreatic cancers

**DOI:** 10.1186/s12885-020-07005-x

**Published:** 2020-06-08

**Authors:** Tomer Boldes, Keren Merenbakh-Lamin, Shani Journo, Eliya Shachar, Doron Lipson, Adva Yeheskel, Metsada Pasmanik-Chor, Tami Rubinek, Ido Wolf

**Affiliations:** 1 Institute of Oncology, Tel Aviv Sourasky Medical Center, 6423906 Tel Aviv, Israel; 2 Sackler Faculty of Medicine, Tel Aviv University, 6997801 Tel Aviv, Israel; 3 Foundation Medicine, Inc., Cambridge, MA 02141 USA; 4 The Bioinformatics Unit, George S. Wise Faculty of Life Sciences, Tel Aviv University, 6997801 Tel Aviv, Israel

**Keywords:** Pancreatic cancer, Estrogen receptor, Activation Function-1, SNP

## Abstract

**Background:**

Estrogen receptor α (ESR1) plays a critical role in promoting growth of various cancers. Yet, its role in the development of pancreatic cancer is not well-defined. A less studied region of ESR1 is the hinge region, connecting the ligand binding and DNA domains. rs142712646 is a rare SNP in ESR1, which leads to a substitution of arginine to cysteine at amino acid 269 (R269C). The mutation is positioned in the hinge region of ESR1, hence may affect the receptor structure and function. We aimed to characterize the activity of R269C-ESR1 and study its role in the development of pancreatic cancer.

**Methods:**

Transcriptional activity was evaluated by E2-response element (ERE) and AP1 –luciferase reporter assays and qRT-PCR. Proliferation and migration were assessed using MTT and wound healing assays. Gene-expression analysis was performed using RNAseq.

**Results:**

We examined the presence of this SNP in various malignancies, using the entire database of FoundationOne and noted enrichment of it in a subset of pancreatic non-ductal adenocarcinoma (*n* = 2800) compared to pancreatic ductal adenocarcinoma (PDAC) as well as other tumor types (0.53% vs 0.29%, *p* = 0.02). Studies in breast and pancreatic cancer cells indicated cell type-dependent activity of ESR1 harboring R269C. Thus, expression of R269C-ESR1 enhanced proliferation and migration of PANC-1 and COLO-357 pancreatic cancer cells but not of MCF-7 breast cancer cells. Moreover, R269C-ESR1 enhanced E2-response elements (ERE) and AP1-dependent transcriptional activity and increased mRNA levels of ERE and AP1-regulated genes in pancreatic cancer cell lines, but had a modest effect on MCF-7 breast cancer cells. Accordingly, whole transcriptome analysis indicated alterations of genes associated with tumorigenicity in pancreatic cancer cells and upregulation of genes associated with cell metabolism and hormone biosynthesis in breast cancer cells.

**Conclusions:**

Our study shed new light on the role of the hinge region in regulating transcriptional activity of the ER and indicates cell-type specific activity, namely increased activity in pancreatic cancer cells but reduced activity in breast cancer cells. While rare, the presence of rs142712646 may serve as a novel genetic risk factor, and a possible target for therapy in a subset of non-ductal pancreatic cancers.

## Background

The human estrogen receptor α (ERα), encoded by *ESR1*, is a member of the steroid/nuclear receptor superfamily and functions as ligand-activated transcription factors [1]. Upon binding of estrogen, the ER dimerizes and binds to coactivators. The complex is then recruited to the estrogen-responsive elements (ERE) on the promoters of ER target genes. The major functional domains of ERα are the N-terminal Activation Function-1 (AF-1) which modulates transcription, the DNA-binding domain (DBD) and the ligand-binding domain (LBD) that contains Activation Function-2 (AF-2) [2]. A less characterized domain of ERα is the hinge region, which lies between the DBD and the LBD. The hinge region contains putative nuclear localization sequence (NLS) and may play a role in transcriptional regulation [1, 3–6].

Approximately 75% of all breast cancers express ERα, and targeting ERα signaling, is a key treatment strategy in these tumors. ERα plays a major role in the development of other malignancies, including endometrial and ovarian cancers [7] and is expressed in subsets of additional tumors, including lung [8] gastric [9] and colon [10] cancers. Pancreatic cancer is the fourth leading cause of cancer death, with five-year survival of roughly 8% [11]. Known risk factors for pancreatic cancer include smoking, diabetes, obesity and pancreatitis [12] and up to 10% of the cases are attributed to high-risk inherited mutations [13, 14]. Yet, initiating genetic mechanisms leading to the development of pancreatic cancer are mostly unknown. Pancreatic ductal adenocarcinoma (PDAC) is the most common histological subtype of pancreatic cancer, representing 85% of all pancreatic neoplasms while less common subtypes include adenosquamous, mucinous, anaplastic and signet ring cancers [15]. Interestingly, ERα is also expressed in a subset of pancreatic adenocarcinoma, most notably in mucinous tumors [16–19] and in vitro and in vivo studies indicated growth inhibition of pancreatic cancer cells by tamoxifen [20, 21]. Several clinical trials reported on activity of hormonal therapy in pancreatic cancer [22–28] including a prospective randomized trial that reported on median survival of 5.3 months among 37 tamoxifen-treated patients, compared to 3 months in 39 patients treated with a placebo, with marginal statistical significance (*p* = 0.07) [29]. Yet, no benefit was noted in a smaller trial [30].

A documented rare SNP in the ESR1 gene (rs142712646) leads to a substitution of arginine at position 269 of the hinge region to cysteine (R269C). To our knowledge, the activity of this SNP, and its potential role in tumorigenesis, has not been reported yet. We report here on enrichment of this functional variant in non-PDAC pancreatic cancers. In this study, we aimed to characterize the activity of R269C-ESR1 in pancreatic and breast cancer cells and identify its role as a potential driver of proliferation of pancreatic cancer cells. Functional analysis revealed increased AP-1 dependent gene-expression of this variant in pancreatic but not in breast cancer cells, and expression of the R269C variant enhanced proliferation and migration of pancreatic cancer cells. These data indicate unique, cell-type dependent activity, of R269C and its contribution to tumor aggressiveness in a small subset of pancreatic cancers.

## Methods

### Foundation medicine database analysis

We searched for mutations in the ESR1 gene, in the entire database of the Foundation One clinical database (Foundation One, Foundation Medicine, Cambridge, MA). The database consists of > 100,000 cases, of them > 4000 PDAC cases and ~ 2800 non-PDAC pancreatic cancer. The test has been described previously [31] and consists of deep sequencing of cancer-related genes on DNA extracted from paraffin embedded tissue samples.

### Computational structure analysis

The secondary structure of ERα was predicted using ConSSert [32], PsiPred [33], Jnet [34] and Predator [35]. A multiple sequence alignment of Human ERα and other 38 vertebrates homologs, collected from SwissProt, was calculated using Mafft [36]. The alignment and secondary structure annotations were presented in Jalview [37]. Known domains of ERα were taken from Pfam [38].

### Reagents and antibodies

17β-Estradiol (E_2_) and crystal violet were obtained from Sigma (St. Louis, MO); ICI 182,780 from Tocris Bioscience (Ellisville, MO), G418 from Life Technologies (Waltham, MA); qScript cDNA SuperMix and PerfeCTa SYBR Green FastMix from Quanta BioSciences (Gaithersburg, MD). Primers synthesis- IDT (Coralville, IA).

### Plasmids and constructs

The ERE-luciferase reporter construct, kindly provided by D. Harris, (UCLA, CA), consists of 2 repeats of the upstream region of the vitellogenin ERE promoter. pRL Renilla luciferase control was purchased from Promega (Cat no E2261, Promega, Madison, WI). The generation of WT-ER construct (in pcDNA3) was described elsewhere [39]. Arginine to cysteine mutation (R269C-ER) was inserted using WT-ER as a template (generated by GeneScript Inc. HK, China).

### Cells and transfection

Human kidney cell line HEK293, breast cancer cell line MCF-7 and pancreatic cancer cell lines COLO-357 and PANC-1 were obtained from ATCC (Manassas, VA). All cells were maintained in Dulbecco’s modified Eagle’s medium (DMEM), containing 10% fetal bovine serum and 100 U/ml penicillin/streptomycin (1%) at 37 °C in a humidified 5% CO2 atmosphere. All experiments were conducted with cells under 15 passages. For estrogen studies, cells were cultured in phenol-free media using 10% charcoal-treated serum (Beit Haemek, Israel) for 2 days before treatment. All transfections were conducted with Jet PEI (Polypus Transfection, Illkirch, France) according to the manufacturer’s instructions.

### Luciferase assays

The assays were conducted essentially as described [40–42]. In brief, cells grown in phenol-free media using 10% charcoal-treated serum were plated in 96-well plates, and transiently transfected with the constructs (WT-ER or R269C-ER), reporter vector (ERE-luciferase or AP-1 luciferase) and Renilla vector. Twenty-four hours later cells were treated with 10 nM E2 or a 0.0003% ethanol as a control vehicle for the ERE-luciferase assay or with ICI 182780 or 0.001% DMSO as a control vehicle for the Ap-1-luciferase assay [43]. At indicated times Dual-Glo Luciferase (Promega) reagent was added to the medium then the cells were incubated at 25 °C for 30 min afterwards the firefly luminescence was measured by multichannel plate spectrophotometer. After the first reading, Dual-Glo® Stop & Glo (Promega) reagent was added to the plate, cells were incubated at 25 °C for 30 min, then Renilla luminescence was measured similarly. Luciferase activity was normalized by calculating the ratio of Firefly to Renilla luciferase units.

### Quantitative real time reverse transcription-PCR (qRT-PCR)

Two days after transfection with the different constructs, total RNA was prepared using the High Pure RNA Isolation Kit Roche (Roche). Total RNA (1 μg) was reverse transcribed using qScript cDNA synthesis kit (Quanta Biosciences). Quantitative RT-PCR (qRT-PCR) was used to determine mRNA level. Primers were designed using Primer Express (Applied Biosystems, Foster City, CA, USA) and synthesized by IDT (Coralville, IA). Primers used: GREB1a (human): F 5′-ACGTGTGGTGACTGGAGTAGC, R 5′- CCACGCAAGGTAGAAGGTGA; TGF-α (human): F 5′- CCCGCTGAGTGCAGACC, R 5′-ACGTACCCAGAATGGCAGAC; CyclinD-1 (human): F 5′- TGGAGGTCTGCGAGGAACAG, R 5′- AGCTGCAGGCGGCTCTTT; IGF-1R (human): F 5′- ATGTCCAGGCCAAAACAGGAT, R 5′- CAACCCTCCCACGATCAACA. Equal loading was determined using β-actin–specific primers. Amplification reactions were performed with Platinum qPCR SuperMix in triplicate using StepOne Plus (Applied Biosystems). PCR conditions: 50 °C for 2 min, 95 °C for 2 min, followed by 40 cycles of 95 °C for 15 s, 60 °C for 45 s.

### Migration assay

Migration was assessed using the wound-healing (“scratch”) assay. COLO-357, PANC-1 and MCF-7 cells were grown to confluency in 6-well plates, with the various constructs (pcDNA3, WT-ER or R269C-ER) and grown in phenol-free media with 10% charcoal-treated serum for 24 h. Cells’ monolayer was scraped in a straight line with a 200 μL sterile pipette tip for 48 h. The cells were photographed at 0, 24, and 48 h with an inverted phase-contrast microscope (Olympus, Tokyo, Japan). Calculation of cell migration (d) was determined using the equation $=\frac{m1-m2}{2}$, when m1 is wound width at time 0 and m2 at 24 or 48 h.

### Western blot analysis

Cells were washed twice with PBS, snap frozen in liquid nitrogen and stored at − 80 °C until the analysis. Cells were harvested and lysed for total protein extraction in radio-immunoprecipitation assay (RIPA) buffer (50 mM Tris-HCl, pH 7.4, 150 mM NaCl, 1% NP-40, 0.25% Na-deoxycholate, 1 mM EDTA, 1 mM NaF) together with a protease inhibitor cocktail (Sigma). A total of 50 μg protein extracts were loaded on 10% polyacrylamide gels, separated electrophoretically and blotted from the gel onto nitrocellulose membrane (Schleicher & Schuell Bioscience GmbH, Dassel, DE, USA). The membranes were blocked with skim milk 1% in PBS-T (0.01 M Tris-HCl pH -7.6, 0.15 M NaCl, 0.2% Tween 20) for an hour, and then immunoblotted overnight with the indicated antibodies. β-actin antibody was used as loading control. Membranes were washed 5 times with TBS-T, followed by incubation with horse raddish peroxidase (HRP, Jackson Immuno Research, West Grove, PA) conjugated antibodies and were detected by using enhanced chemiluminescence (ECL) reaction.

### Proliferation assay

3-(4,5-dimethylthiazol-2-yl)-2,5-diphenyltetrazolium bromide (MTT) assay was conducted essentially as described [44]. In brief, cells were counted and plated in 96-well plates (5000 cells/well), and transiently transfected with WT-ER, R269C-ER or an empty vector then cultured in phenol-free media with 10% charcoal-treated serum. Twenty-four hours later cells were treated with E2 (10 nM) or 0.0003% ethanol as a control vehicle and at indicated time points MTT reagent was added to the medium (500 μg/ml). Cells were incubated for 1.5 h, afterward medium was removed, 100% DMSO was added and absorbance was determined at a wavelength of 570 nm using a multichannel plate spectrophotometer.

### RNAseq

MCF-7 or COLO-357 cells were seeded in phenol red depleted medium with charcoal stripped serum. Cells then were transfected with either WT-ER or R269C-ER in triplicates and then treated with vehicle control or E2 (10nM) for 24 h. Total RNA was extracted using the High Pure RNA Isolation Kit (Roche, Mannheim, Germany). RNAseq and bioinformatics were conducted at the Tel-Aviv University Genomics Research Unit and Bioinformatics Unit (Tel-Aviv, Israel). The libraries were prepared using NEBNext® Ultra™ II RNA Library Prep Kit for Illumina® (New England Biolabs®inc., 240 County Road Ipswich, MA). For sequencing: briefly, 1000 ng of total RNA were fragmented followed by reverse transcription and second strand cDNA synthesis. The double strand cDNA was subjected to end repair, A-base addition, adapter ligation and PCR amplification to create barcoded libraries. Libraries were evaluated by Qubit and TapeStation. Sequencing was conducted with NextSeq 500/550 v2.5 (Illumina) at 75-cycles, Single Read kit. The output was ~ 21 million reads per sample.

### Bioinformatics

Poly-A/T stretches and Illumina adapters were trimmed from the reads using cutadapt; resulting reads shorter than 30 bp were discarded. FastQ files were uploaded to Partek Flow [45] for processing. Reads were mapped to the *Homo sapiens* GRCm38 reference genome using BWA-MEM [46] (with default parameters). Quantification to annotation model was performed using Partek E/M [47] and RPKM normalized expression levels for each gene were obtained. Differentially expressed genes were identified using GSA (Genome specific analysis) [48, 49]. Pathway and function enrichment were analyzed using specified web-tools and heatmaps of genes associated with specific pathways were generated using Partek Genomic Suite [50].

### Statistical analysis

Each experiment was performed at least three times. The data were expressed as the mean ± SD or SE. Statistical significance was assessed by Student’s t test. A *P* value of < 0.05 is considered statistically significant.

## Results

### Prevalence of R269C substitution in *ESR1* in clinical samples of pancreatic cancer

The substitution of C to T at position 1039, leading to a substitution of arginine at position 269 to cysteine (R269C), was observed in a patient with mucinous pancreatic adenocarcinoma conducting tumor genomic analysis for clinical purposes. This substitution is a known rare SNP (rs142712646) [51] and its frequency among European population is estimated to be 0.14% according to the ExAC dataset. Following this observation, the frequency of this alteration was examined on the entire clinical database of FoundationOne, containing > 150,000 tumor samples of various origins. The observed frequency in a wide range of malignancies, including breast and pancreatic ductal adenocarcinoma, was 0.29% and was considered to be similar to the frequency at the ExAC dataset. Yet, a significantly higher frequency was noted in ~ 2800 non-PDAC pancreatic cancer (0.53%, *p* = 0.02). This enrichment suggests that the R269C substitution may play a role in the development of these tumors.

### Structural analysis of ESR1 harboring the R269C alteration

**Fig. 1:**
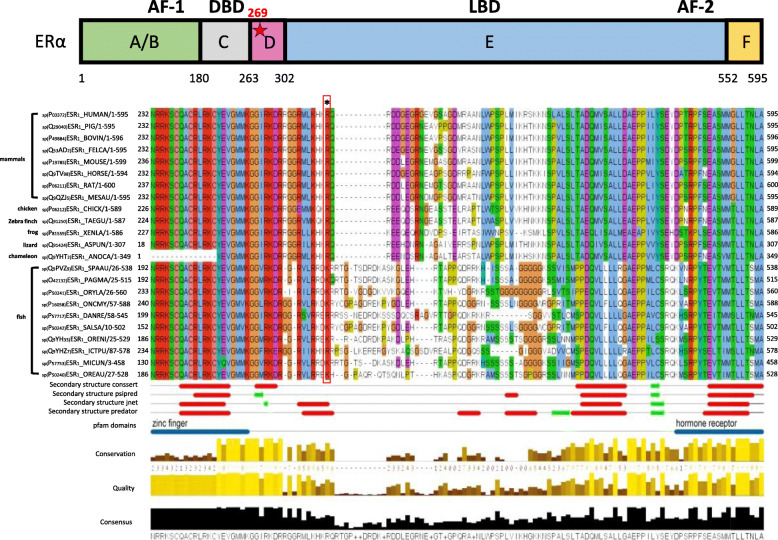
Position 269 is conserved as arginine or lysine among species. Multiple Sequence Alignment (MSA) of ESR1 39 sequences, colored by amino acid type. The mutated position is marked by asterisk above the alignment. Four different secondary structure prediction methods are shown below the alignment with red and green segments (red symbolizes an alpha helix and green- beta strand). The Pfam domains are shown by blue lines. The conservation of the residues is shown by brown-yellow bars. The higher and yellow bars indicate highly conserved positions. The figure was generated using Jalview. Position 269 is conserved as arginine (R) or lysine (K) among species as can be seen in the red rectangle. It is located at the end of a possible alpha helix, in a region connecting a zinc finger domain and the hormone receptor domain

R269 lies within a potential nuclear localization sequences (NLS), known as p-NLS2 [52, 53]. Analysis of Swissprot database indicated position 269 to be highly conserved among species as either arginine or lysine (Fig. 1). Structural analysis using four different secondary structure prediction tools (described under Materials and Methods) suggests that it is located at the end of a possible α-helix, in a region connecting a zinc finger domain and the hormone binding domain. Thus, R269 is predicted to be in contact with residues from the DNA binding domain and the steroid binding domain, and as arginine (pKa = 12.5) is positively charged at physiological pH, the substitution to cysteine would result in a change from a positive charge to a more negative one at position 269. Moreover, it may lead to the formation of wrong SS bonds in the zinc finger domain, containing several conserved cysteine residues.

### Increased estrogen-dependent transcriptional activity of R269C-ER compared to WT-ER in breast and pancreatic cancer cells

**Fig. 2:**
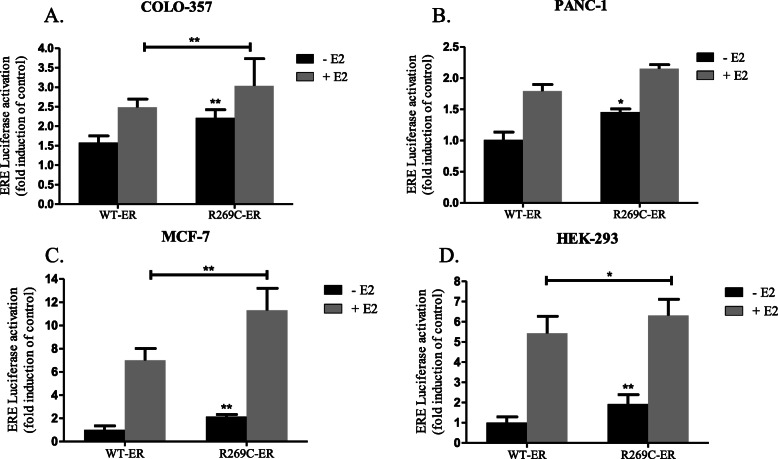
Transcriptional activity of R269C-ER in breast and pancreatic cancer cells. Cells were transiently transfected with either WT-ER or R269C-ER vectors together with the ERE luciferase reporter and treated with E2 (10nM) or a control vehicle for 24 h. Luciferase activity were analyzed and normalized to Renilla luciferase units and are shown relative to the control WT-ER. The results are from a representative experiment of at least three independent experiments, each performed in hexaplicates. Each bar represents the mean ± SD. *, *P* < 0.05, **, *P* < 0.01

In order to study the transcriptional activity of R269C-ER, we generated ER harboring the R269C substitution. Expression of R269C-ER in pancreatic cancer cells was similar to that of overexpressed WT-ER (Supplementary Fig. S1A, B, Additional file 1). The vitellogenin-based ERE-luciferase reporter was then used to study its transcriptional activity, compared to the WT-ER [40]. For the analysis, MCF-7 breast cancer cells, COLO-357 and PANC-1 pancreatic cancer cells and the non-cancerous HEK-293 cells were co-transfected with the reporter and either WT-ER or R269C-ER, grown in estrogen-depleted medium and treated with either vehicle control or E2. R269C-ER demonstrated significantly increased ERE activity compared to WT-ER in all four cell lines, either with or without E2 treatment (Fig. 2, panels a-d). In COLO-357 cells, R269C-ER increased ERE activity by 41% compared to WT-ER and with E2 treatment the mutant increased it by 22% (Fig. 2a, *p* < 0.01). In PANC-1 cells the mutant-ER activity was increased by 44% compared to WT-ER (Fig. 2b, *p* < 0.05). In MCF-7 cells, R269C-ER increased the activity, with or without E2 treatment, by 61 and 114%, respectively (Fig. 2c, *p* < 0.01). Finally, in HEK-293 the mutation increased the activity by 93% (Fig. 2d, *p* < 0.01) and by only 16% with E2 treatment (Fig. 2d, *p* < 0.05).

**Fig. 3:**
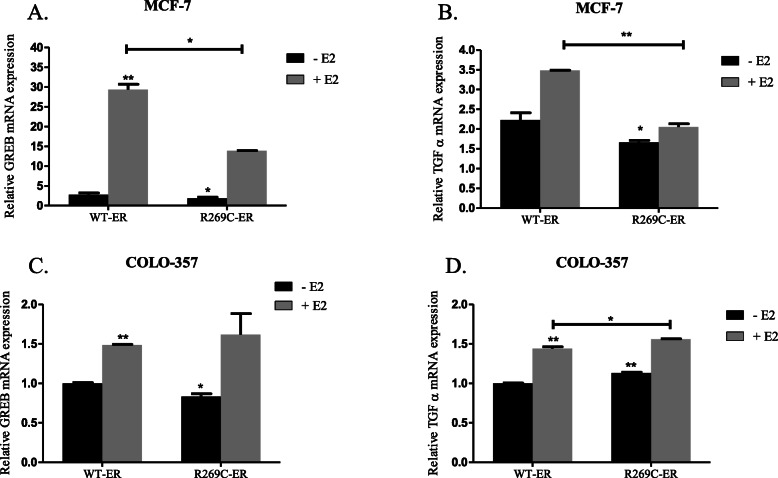
Transcriptional regulation of ER-regulated genes by R269C-ER in breast and pancreatic cancer cells. MCF-7 **(a**, **b)** and Colo-357 cells **(c**, **d)** were transiently transfected with either WT-ER or R269C-ER constructs and treated with E2 (10nM) or a control vehicle for 24 h. mRNA levels of GREB-1 and TGF-α were determined 48 h after transfection by quantitative RT-PCR and are shown relative to the control WT-ER. The results are from a representative experiment of at least three independent experiments, each performed in triplicates, each bar represents the mean ± SD. *, *P* < 0.05, **, *P* < 0.01

In order to further validate the transcriptional activity of R269C-ER, we examined its ability to induce transcription of the classic estrogen-regulated genes GREB1 and TGF-α, which their promoter contains ERE sequences [40]*.* Surprisingly, while overexpression of R269C-ER in MCF-7 cells decreased mRNA levels of GREB1 by 31%, and TGF-α by 25%, and also significantly reduced the response to E2 treatment (Fig. 3a, b, *p* < 0.05 for all comparisons), it enhanced GREB and TGF-α mRNA levels in COLO-357 cells in response to E2 (Fig. 3c, d, *p* < 0.01). These data indicate complex, cell-type dependent transcriptional activity of both WT-ER and R269C-ER in pancreatic cancer cells.

### R269C-ER enhances AP-1 dependent transcriptional activity in breast and pancreatic cancer cells

**Fig. 4:**
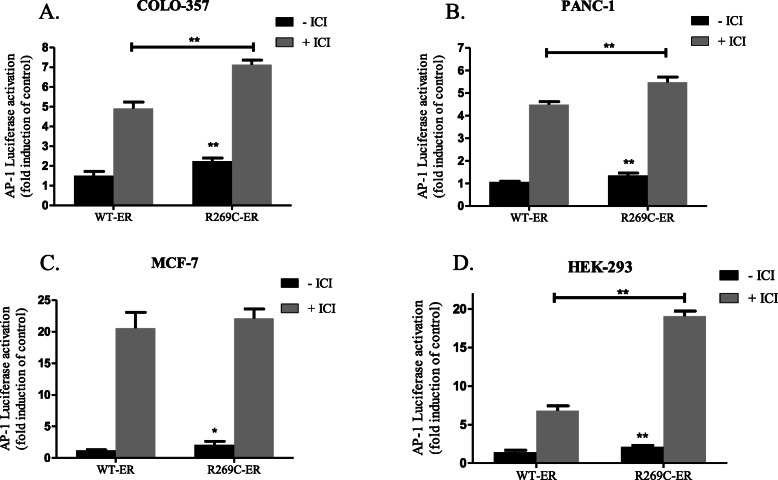
AP-1 dependent transcriptional activity of R269C-ER. Cells were transiently transfected with either WT-ERα or R269C-ERα constructs together with the AP-1 luciferase reporter and treated with ICI 182780 (100 nM) or a control vehicle for 24 h. Luciferase activities were analyzed and normalized to Renilla luciferase units and are shown relative to the control WT-ER. The results are from a representative experiment of at least three independent experiments, each performed in hexaplicates, each bar represents the mean ± SD. *, *P* < 0.05, **, *P* < 0.01

The hinge region has been suggested to mediate non-classical transcription through interaction with the AF-1 domain and with transcription factors regulating AP-1 activity (e.g. c-Fos/c-Jun, Sp1). In order to analyze the effects of R269C-ER on AP-1 activity, an AP-1-luciferase reporter was employed [41]. COLO-357, PANC-1, MCF-7 and HEK-293 cells were co-transfected with the AP-1-reporter and either WT-ER or R269C-ER. Cells were grown in estrogen-depleted medium with or without fulvestrant (ICI 182,780), which due to its AP-1 agonist activity, served as a positive control [42, 43, 54]. In comparison to WT-ER, in COLO-357 cells, R269C-ER increased AP-1 transcriptional activity by 48%, in PANC-1 cells by 27%, in MCF-7 cells by 74% and in HEK-293 cells by 49% (Fig. 4a-d, *p* < 0.05 for all comparisons).

**Fig. 5:**
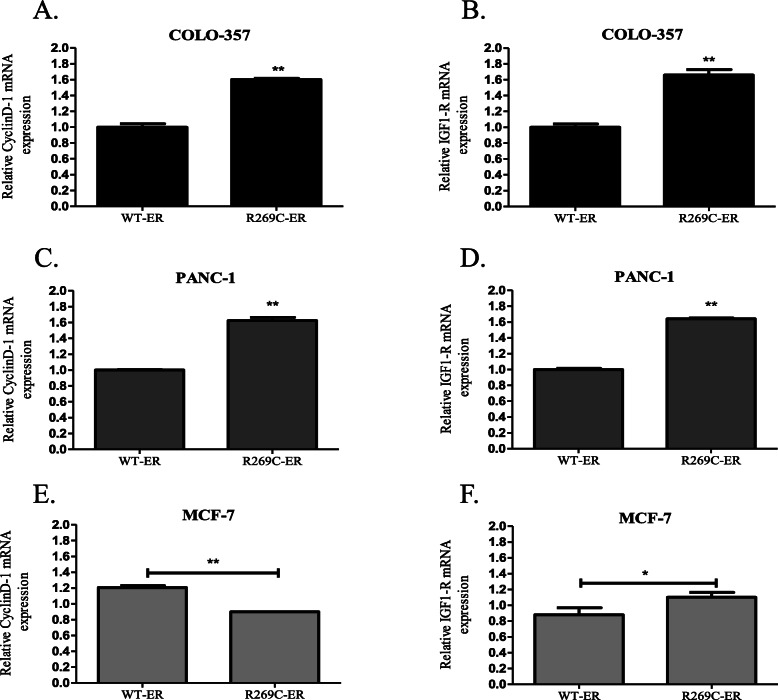
Transcriptional regulation of AP-1-regulated genes in breast and pancreatic cancer cells by R269C-ER. COLO-1 cells **(a**, **b)** PANC-1 cells **(c**, **d)** and MCF-7 cells **(e**, **f)** were transiently transfected with either WT-ER or R269C-ER constructs and treated with a control vehicle for 24 h. mRNA levels of cyclin D1 and IGF1-R were determined 48 h after transfection by quantitative RT-PCR. The results are from a representative experiment of at least three independent experiments, each performed in triplicates, each bar represents the mean ± SD. *, *P* < 0.05, **, *P* < 0.01

Next, the effect of R269C-ER on the transcription of the AP-1-regulated genes was examined. Expression of R269C-ER in COLO-357 cells increased mRNA levels of both cyclin D1 and IGF-1R by 60 and 65%, respectively (Fig. 5a-b, *p* < 0.01). Similarly, in PANC-1 cells we observed an increase of cyclin D1 and IGF-1R mRNA by 64 and 62%, respectively (Fig. 5c-d, *p* < 0.01). While statistically significant, the effect of R269C-ER on MCF-7 cells was less pronounced: it decreased the levels of cyclin D1 by 25%, and increased levels of IGF-1 by 25% (Fig. 5e-f, *p* < 0.05). Taken together, these data indicate AP-1 mediated transcriptional activity of R269C-ER, which is more profound in pancreatic cancer cells compared to breast cancer cells.

### Global transcriptomic analysis reveals cell-type dependent effect of R269C-ER in breast and pancreatic cancer cells

**Fig. 6:**
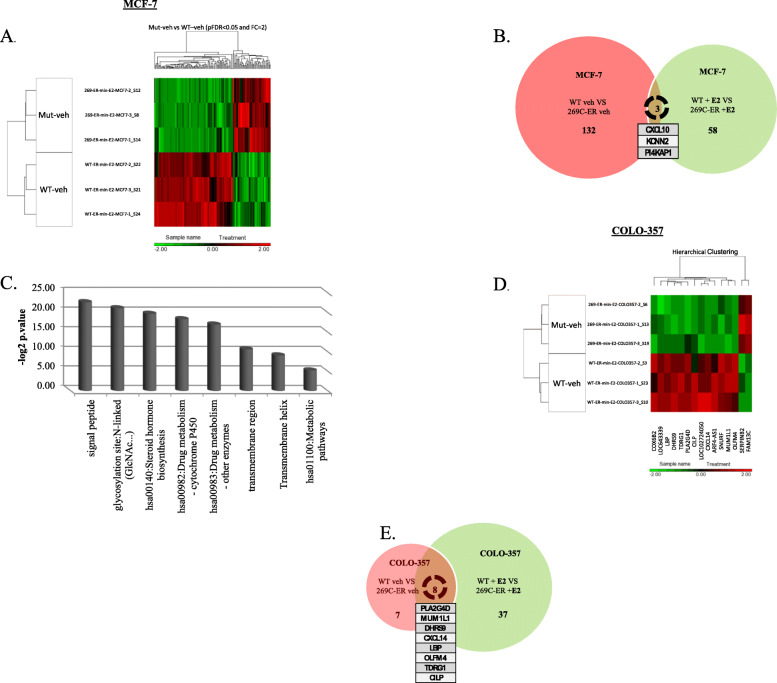
R269C-ER exhibits differential effect on gene expression in pancreatic cancer cells compared to breast cancer MCF-7 and COLO-357 cells were seeded in phenol red depleted medium with charcoal stripped serum. Cells then were transfected with either WT-ER or R269C-ER in triplicates and then treated with vehicle control or E2 (10nM) for 24 h. Total RNA was extracted and RNAseq was performed. **a** A heatmap of differentially expressed genes, between untreated mutated and WT-ER, in MCF-7 cells was generated. **b** A Venn-diagram of significantly (*p* < 0.05) upregulated or downregulated genes in MCF-7 compared to MCF-7 cells treated with E2 is depicted. **c** Pathway enrichment analysis was conducted in MCF-7 cells treated with E2 (10nM), using Gene Analytics™. Results show that R269C-ER overexpression is associated with different metabolic pathways. **d** A heatmap of differentially expressed genes, between untreated mutated and WT-ER, in COLO-357 cells was generated **e** A Venn-diagram of significantly (*p* < 0.05) upregulated or downregulated genes in COLO-357 compared to COLO-357 treated with E2 is depicted

**Fig. 7:**
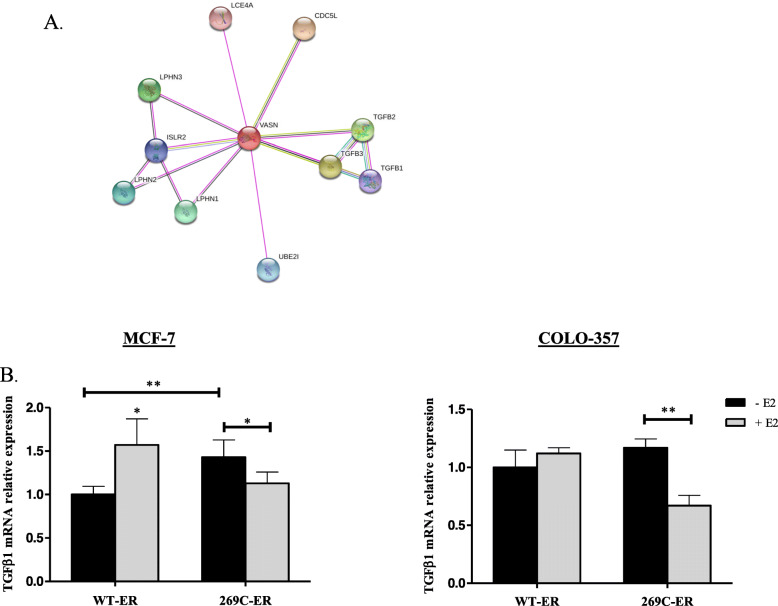
R269C-ER decreases TGFβ expression in pancreatic cancer. **a** A string analysis of VASN gene is depicted. **b** MCF-7 and COLO-357 cells were seeded in phenol red depleted medium with charcoal stripped serum. Cells then were transfected with either WT-ER or R269C-ER in triplicates and treated with vehicle control or E2 (10nM) for 24 h. Total RNA was extracted and expression of TGFβ1 was assessed. Each graph represents ± SD. *, *P* < 0.05, **, *P* < 0.01. Representative experiment is shown

In order to further delineate the transcriptional activity of 269C-ER, a global transcriptomic analysis of MCF-7 and COLO-357 cells expressing either R269C-ER or the WT-ER was performed. Compared to WT-ER, expression of R269C-ER in MCF-7 resulted in differential regulation of 135 genes (44 upregulated, 91 downregulated) with most upregulated genes associated with cell metabolism and hormone biosynthesis (Fig. 6a, b). Interestingly, when treated with E2, an independent set of 61 genes was differentially regulated, with only 3 genes shared with untreated cells (Fig. 6b). Similar analysis of COLO-357 cells indicated differential regulation of 15 and 45 genes in untreated and E2-treated cells, respectively, with eight genes common to the two groups (Fig. 6d, e). Importantly, genes regulated by R269C-ER in COLO-357 cells were associated with a more aggressive behavior (Fig. 6e). As we focused on the effect of R269C-ER on pancreatic cancer, we searched for genes known to play a role specifically in this type of cancer. We observed a 6-fold decrease in VASN gene expression (Fig. 2S), a gene involved in the TGFβ pathway, as evidenced by String analysis (Fig. 7a). Thus, we assessed TGFβ1 mRNA expression and found it decreased in R269C-ER expressing cells upon E2 treatment compared to WT-ER (*p* < 0.01, Fig. 7b). As TGFβ1 may play suppressive role in pancreatic cancer development [55, 56], inhibition of this pathway, may be a possible mechanism by which R269C-ER mediates aggressive behavior of pancreatic cancer cells.

### R269C-ER enhances migration of pancreatic but not of breast cancer cells

**Fig. 8:**
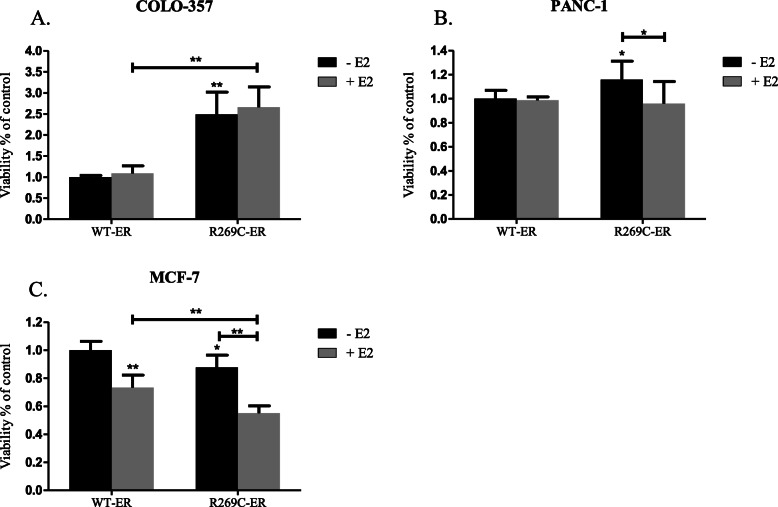
Cell-type dependent effects of R269C-ER on cancer cell proliferation. COLO-357 (**a**), PANC-1 (**b**) and MCF-7 (**c**) cells were transiently transfected with either WT-ER or R269C-ER, seeded in 96-well plates and grown in estrogen-depleted medium and after 24 h were treated with either E2 (10nM) or a vehicle control for 72 h. Viability was assessed using MTT assay and are shown relative to the control WT-ER. Figures show representative results of at least three independent experiments, each performed in hexaplicates, each bar represents the mean ± SD. *, *P* < 0.05, **, *P* < 0.01

The effects of R269C-ER on proliferation of MCF-7, COLO-357 and PANC-1 cells were evaluated using MTT assay. For the analysis, cells were transfected with WT-ER or R269C-ER, grown in estrogen-depleted medium, and treated with either a vehicle control or E2. While R269C-ER enhanced proliferation of COLO-357 in the basal state by 149% (Fig. 8a, *p* < 0.01) and in PANC-1 by 16% (Fig. 8b, *p* = 0.03). In contrary, it significantly decreased proliferation of MCF-7 cells by 12% (Fig. 8c, *p* = 0.02).

**Fig. 9:**
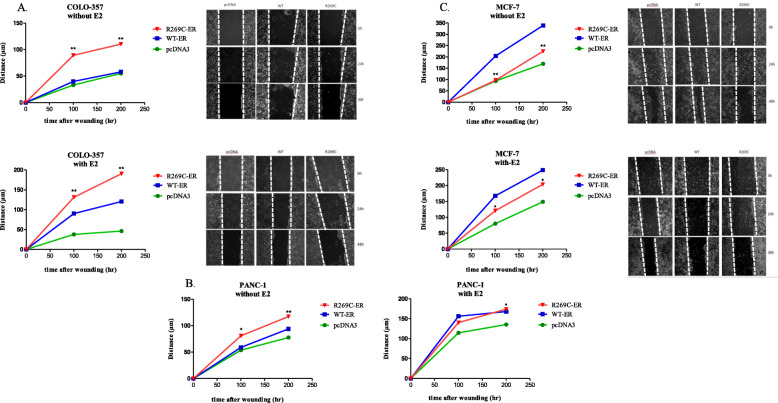
R269C-ER enhances migration of pancreatic cancer cells. COLO-357 (**a**) PANC-1 (**b**) and MCF-7 (**c**) were seeded in 6-well plates and transfected with either WT-ER, R269C-ER or pcDNA3 constructs. Cells were grown in estrogen-depleted medium and the monolayer was scraped in a straight line, then treated with E2 (10nM) or a vehicle control for 48 h. The results are from a representative experiment of at least three independent experiments. Mean values of at least 10 measurements for each time point and condition are shown on the graphs and representative photos are also presented, *, *P* < 0.05, **, *P* < 0.01

The ability of R269C-ER to enhance migration was assessed by wound healing assay. Expression of R269C-ER significantly enhanced migration of COLO-357 cells compared to WT-ER both after 24 h and 48 h by 123 and 90%, respectively, and with E2 treatment by 45 and 58%, respectively (Fig. 9a, *p* < 0.01). Similar results were observed with E2-untreated PANC-1 cells, after 24 h the migration was enhanced by 37% and after 48 h by 25% (Fig. 9b, *p* < 0.01 and *p* < 0.05, respectively). However, the mutation did not increase migration of MCF-7 cells, but rather slowed migration compared to WT-expressing cells (Fig. 9c).

## Discussion

While early works suggested a possible role for ER-α in the development of pancreatic cancer [16–19, 57, 58], its role in the development of this cancer is still controversial. To our knowledge, this is the first report to identify a possible link between this rare variant of the ER and pancreatic cancer development. While this variant is not common, the possibility of treating even a small subset of pancreatic cancer patients with hormonal therapy, which is definitely less toxic and possibly more effective than standard chemotherapy, may be of great value to patients. In addition, our study highlights for the first time, mechanistic aspects of ER activation related to the structure of the hinge region, a much less studied region of the ER, and suggests a role for this region in mediating AP-1 transcriptional activity.

We sought to examine the prevalence and activity of R269C-ER, a rare functional variant of ESR1, in breast and pancreatic cancers. Analysis of a large genomic database indicated enrichment of this variant in a small subset of non-PDAC pancreatic cancers, and functional analysis suggested cell-type dependent activity of this variant. Thus, it showed increased classic and AF-1-mediated transcriptional activity specifically in pancreatic cancer cells while less in MCF-7 breast cancer cells and accordingly enhanced proliferation and migration only of pancreatic cancer cells. A transcriptomic analysis further corroborated these results.

Pancreatic tumors are diverse in histological, molecular and biological features. Both COLO-357 and PANC-1 originate from patients with PDAC, but the phenotype and genotype of those cell lines are different. The phenotype of COLO-357 cells remains unknown [59] while the genotype grade (G) is classified as G1-G2 [60]. For PANC-1 cells the phenotype is depict as ductal/acinar [59], and the genotype is classified as G3 [61]. The features of those cell lines are useful in understanding and evaluating the mutation impact on pancreatic cancer, though should be carefully addressed, as there is much heterogeneity even within the tumor characteristic. Furthermore, we used ER-positive breast cancer cells (MCF-7) for deeper understanding of the mutation and in effort to examine cells that their tumorgenicity depends on the ER pathway.

R269 lies within the hinge region of ESR1, a region that has not been studied extensively. Position 269 is highly conserved across species as arginine or lysine and lies within a putative NLS region. Substitution of arginine (a conserved negative charge) to cysteine is expected to alter its interactions with both AF-1 and AF-2. In silico study via multiple bioinformatics tools (Polyphen-2, SIFT and PROVEAN), predicted with high probability that R269C substitution may damage the structure-function of this protein [62].

In agreement with these predictions, our data indicates altered transcriptional activity of the variant compared to the WT receptor, an effect that was even more pronounced toward AF-1 than toward AF-2. These observations point to an important role of the hinge region in maintaining proper transcriptional activity of the ER and may explain the low frequency of alterations observed in this area in the general population and among species. We noted a discrepancy between direct transcriptional activity of R269C-ER, as determined using the ERE reporter, and the effect on mRNA of ERE-regulated genes in MCF-7 cells. This observation suggests a role for additional, possibly non-genomic mechanisms regulating the activity of R269C-ER specifically in these cells. The mutation lies within putative NLS2 and possibly affects the activity of the receptor by modifying its subcellular localization, however nuclear and cytoplasmic localization were not disrupted compared to WT-ER (data is not shown).

Surprisingly, the effect of R269C variant was cell type specific. It inhibited gene expression, proliferation and migration of MCF-7 cells. The precise upstream regulatory elements leading to these differential effects are yet to be determined. This observation explains the lack of enrichment of R269C among breast cancer patients. Thus, expression of R269C is probably not associated with increased breast cancer risk. On the other hand, expression of R269C had profound effect on pancreatic cancer cells. It enhanced transcriptional activity of both classical and AF-1-regulated genes, enhanced proliferation and promoted migration of two pancreatic cancer cell lines. These observations may explain the enrichment of R269C among a subset of pancreatic cancers. It also highlights the need to study the effects of this mutation in additional pancreatic cancer subtype models.

A global transcriptome analysis, using RNAseq, indicated differential gene expression among breast and pancreatic cancer cell lines expressing the mutated ER. Thus, expression of the R269C-ER was associated with ligand-independent upregulation of genes associated with cell metabolism and hormone biosynthesis, and not with growth or invasion. This observation may explain the lack of association between the presence of this SNP and increase breast cancer risk. On the one hand, alterations of genes associated with tumorogenicity were observed in COLO-357 cells. These included upregulation SERPINB2, a known oncogene [63–65], and a decrease in TGFβ1 expression, a cytokine which may function under specific circumstances as a tumor suppressor in pancreatic cancer [66–68]. Indeed, Hezel et al. showed that TGF-β or αvβ6 blockade increased pancreatic tumor cell proliferation and accelerated both early and later disease stages [55]. Importantly, these observations indicate, for the first time, a direct role of the hinge region in regulating transcriptional activity of the ER.

Previous studies, published mostly during the 1980s and 1990s, explored the role of anti-estrogens as a potential treatment for pancreatic cancer. While pre-clinical studies were promising [69], clinical trials showed conflicting results, partly due to under power of the trials and partly may be attributed to the inclusion of all pancreatic cancer patients, regardless expression of the ER or the presence of functional variants. Importantly, while our study indicated increased activity of this variant, at least part of the increased activity is mediated through the AF-1 and is E2 independent. Therefore, it is likely that current hormonal therapies targeting the ER, either by reducing estrogen levels (aromatase inhibitors) or by inhibition of its classic activity (tamoxifen) may not be effective and other manipulations should be examined. The frequency of the variant, even among non-PDAC patients is low (~ 0.5%). Therefore, the conduction of clinical trials on this specific patients’ population is likely to be very challenging. Yet, considering the grim prognosis and limited treatment options for patients with pancreatic cancer, the ability to target even a small fraction of patients is of major significance.

## Conclusion

Our data suggest a role for R269C functional variant of the ESR1 in the development of a small subset of non-PDAC pancreatic cancers. Moreover, our data suggest that this variant may have a protective effect against breast cancer. The underlying mechanism should be further explored.

## Supplementary information

**Additional file 1: Figure S1.** Expression of R269C-ER in pancreatic cancer cells. PANC-1 **(A)** or COLO-357 **(B)** cells were transfected with either pCDNA3, WT-ER or R269C-ER grown in estrogen-depleted medium and treated with E2 (10nM) or a control vehicle for 24 h. Cells were harvested, lysed and analyzed by Western blotting. The results of **(A)** and **(B)** are from a representative experiment of *n* = 3.

**Additional file 2: Figure S2.** R269C-ER exhibits differential effect following E2 treatment on gene expression in pancreatic cancer cells. A list of genes down and upregulated by R269C-ER compared to WT-ER following E2 treatment in COLO-357 cells.

**Additional file 3: Figure S3.** R269C-ER exhibits differential effect on gene expression in pancreatic cancer cells compared to breast cancer. MCF-7 and COLO-357 cells were seeded in phenol red depleted medium with charcoal stripped serum. Cells then were transfected with either WT-ER or R269C-ER in triplicates and then treated with vehicle control or E2 (10nM) for 24 h. Total RNA was extracted and RNAseq was performed. **(A, B)** A heatmap of differentially expressed genes in MCF-7 cells treated with E2 **(A)** and COLO-357 cells treated with E2 **(B)** was generated.

## Data Availability

The datasets used and/or analyzed during the current study are available from the corresponding author on reasonable request.

## Abbreviations

AF-1: Activation Function-1
AF-2: Activation Function-2
DBD: DNA-binding domain
E_2_: 17β-Estradiol
ECL: Enhanced chemiluminescence
ERE: E2- response elements
ESR1: Estrogen receptor α (ESR1)
G: Grade
HRP: Horse raddish peroxidase
LBD: Ligand-binding domain
MSA: Multiple Sequence Alignment
NLS: Nuclear localization sequence
PDAC: Pancreatic ductal adenocarcinoma
R269C: Substitution of arginine to cysteine at position 269 of the hinge region
RIPA: Radio-immunoprecipitation assay
