# Supplementary figures and images for: R269C variant of ESR1: high prevalence and differential function in a subset of pancreatic cancers
Source: BMC Cancer. 2020 Jun 8;20:531. doi: 10.1186/s12885-020-07005-x (PMC7282172; doi:10.1186/s12885-020-07005-x)

# S1

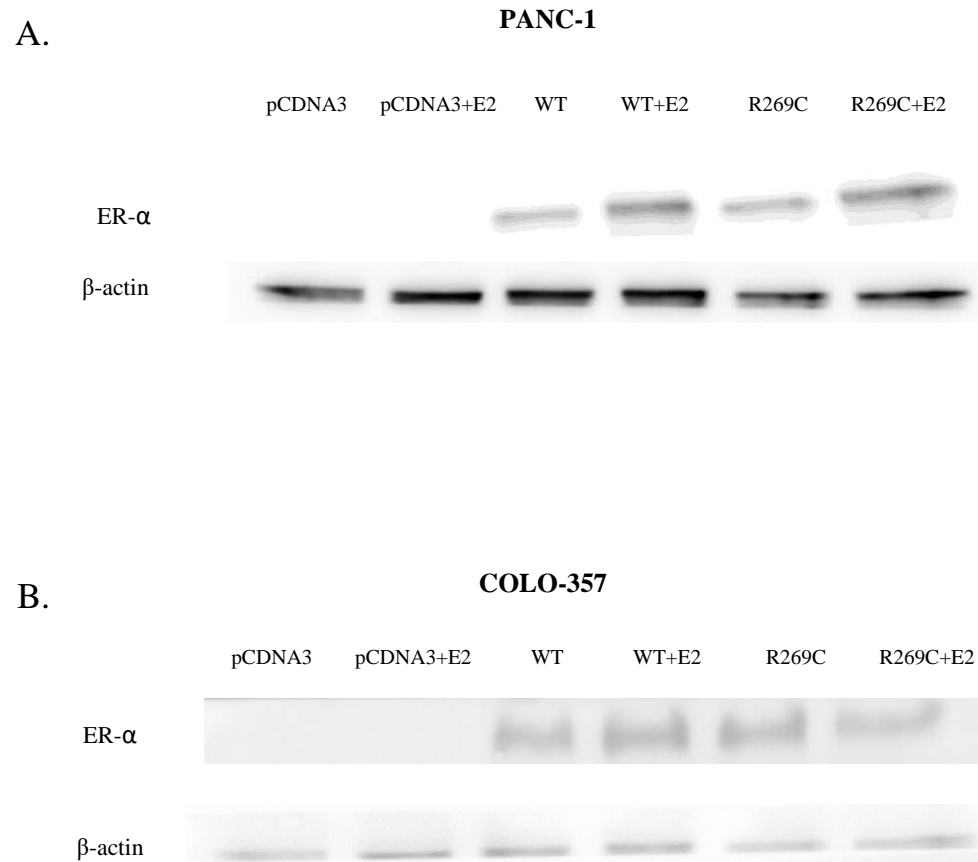

Supplement: Supplementary file 1 — Additional file 1: Figure S1. Expression of R269C-ER in pancreatic cancer cells. PANC-1 (A) or COLO-357 (B) cells were transfected with either pCDNA3, WT-ER or R269C-ER grown in estrogen-depleted medium and treated with E2 (10nM) or a control vehicle for 24 h. Cells were harvested, lysed and analyzed by Western blotting. The results of (A) and (B) are from a representative experiment of n = 3. [file 12885_2020_7005_MOESM1_ESM.pdf]

MCF-7

S3

COLO-357

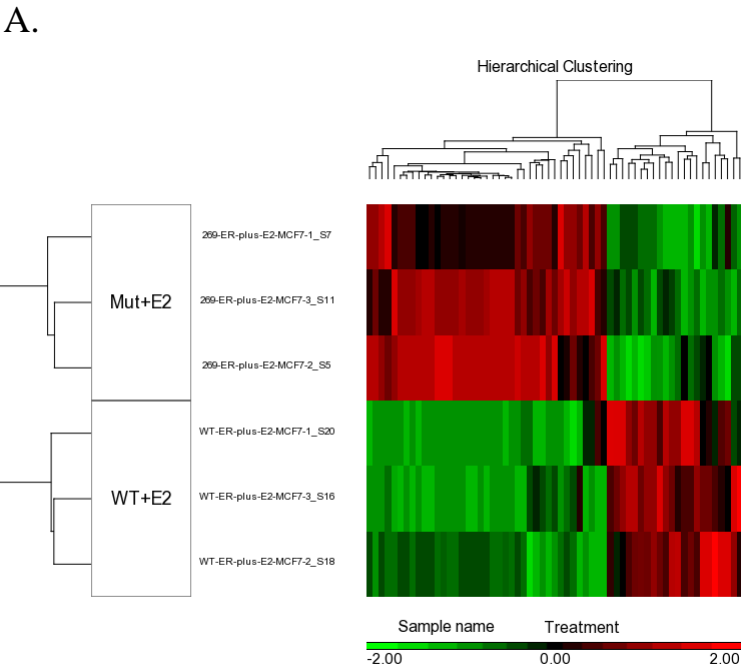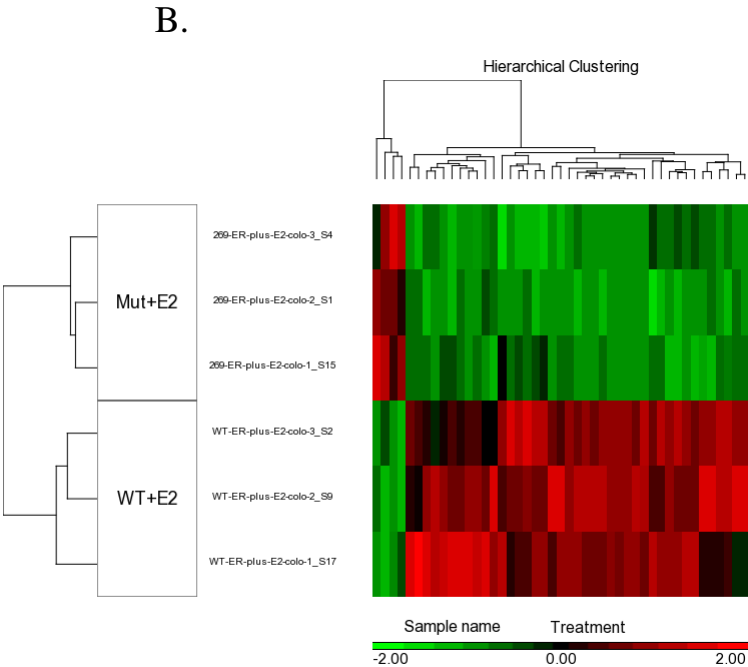

Supplement: Supplementary file 3 — Additional file 3: Figure S3. R269C-ER exhibits differential effect on gene expression in pancreatic cancer cells compared to breast cancer. MCF-7 and COLO-357 cells were seeded in phenol red depleted medium with charcoal stripped serum. Cells then were transfected with either WT-ER or R269C-ER in triplicates and then treated with vehicle control or E2 (10nM) for 24 h. Total RNA was extracted and RNAseq was performed. (A, B) A heatmap of differentially expressed genes in MCF-7 cells treated with E2 (A) and COLO-357 cells treated with E2 (B) was generated. [file 12885_2020_7005_MOESM3_ESM.pdf]
