# Supplementary material for: R269C variant of ESR1: high prevalence and differential function in a subset of pancreatic cancers
Source: BMC Cancer. 2020 Jun 8;20:531. doi: 10.1186/s12885-020-07005-x (PMC7282172; doi:10.1186/s12885-020-07005-x)
Supplement: Supplementary file 2 — Additional file 2: Figure S2. R269C-ER exhibits differential effect following E2 treatment on gene expression in pancreatic cancer cells. A list of genes down and upregulated by R269C-ER compared to WT-ER following E2 treatment in COLO-357 cells. [file 12885_2020_7005_MOESM2_ESM.pdf]

S2

Gene Name	Fold change (269C-ER vs WT-ER)	p-value
LINC00221	9.94936	1.60808E-06
SNORA58	7.39334	0.000264164
LOC105371703	3.218	0.000151507
SLC10A2	3.00466	0.000424669
SPTA1	-2.00505	0.000373446
CHRM4	-2.01712	0.0000460531
ABCB11	-2.0473	3.64666E-08
DUOX2	-2.05579	0.000496647
ITGA11	-2.20964	4.70066E-05
ACMSD	-2.22023	2.53598E-05
LOC642366	-2.22811	0.000479502
KNG1	-2.27623	2.92785E-05
TLR9	-2.34524	0.000440847
GSTM2	-2.40979	0.000344234
LINC00706	-2.4593	0.000111491
MUC17	-2.47735	4.29863E-05
DHRS9	-2.53271	5.10462E-09
CYTH4	-2.62159	0.00030186
LINC01094	-2.6471	0.00026941
SLC51B	-2.77994	4.25982E-11
FGG	-2.84082	5.0933E-07
OLFM4	-3.01867	4.85376E-05
KLRC3	-3.41256	0.000223357
GUCA2B	-3.68262	4.18267E-07
SERPINA1	-3.95624	1.23465E-05
CD79A	-4.06245	0.000297716
OTOF	-4.33232	2.42386E-07
GFRA2	-4.40673	8.89776E-05
PLA2G4D	-4.4699	3.50666E-10
MPPED1	-4.49751	0.000471093
CPN2	-4.80729	0.000438474
PRAP1	-4.92044	0.000417221
MUM1L1	-5.50334	6.63633E-05
CXCL14	-5.83936	7.30615E-14
**VASN**	**-6.03791**	**2.51228E-05**
HCG20	-6.09428	0.000285668
HAMP	-7.28894	4.47808E-05
LBP	-7.63972	9.30596E-12
KCNE1	-7.78223	0.000597159
CILP	-9.73187	4.15455E-13
TDRG1	-10.0774	1.60887E-06
LINC02016	-10.6571	2.94372E-05
B3GNT6	-13.9481	8.73073E-05
SLCO2A1	-15.0136	2.32404E-07
CST1	-53.8649	2.4838E-10
